# Sulphur and Lycopodium

**Published:** 1889-02

**Authors:** 


SULPHUR AND LYCOPODIUM: It is rarely advisable to begin the treatment of a chronic case with Lycopodium, as is well known. The late Dr. Fellger used to say that Lycopodium did not follow Sulphur well in these chronic cases. Have any of our readers had any experience in this connection?—EDITORS.

